# Characterization and Expression of Two Cytoplasmic Phosphoenolpyruvate Carboxykinase Genes Associated with Larval Diapause and Temperature Stress in the Wheat Blossom Midge, *Sitodiplosis mosellana*

**DOI:** 10.3390/biology15020147

**Published:** 2026-01-14

**Authors:** Qitong Huang, Yuxia Nie, Xiaobin Liu, Qian Ma, Wenqian Tang, Weining Cheng

**Affiliations:** 1 Shandong Institute of Sericulture, Shandong Academy of Agricultural Sciences, Yantai 265503, China; 2 Key Laboratory of Plant Protection Resources and Pest Management of Ministry of Education, College of Plant Protection, Northwest A&F University, Yangling 712100, China

**Keywords:** *Sitodiplosis mosellana*, phosphoenolpyruvate carboxykinase, diapause, thermal stress response, larval development

## Abstract

Phosphoenolpyruvate carboxykinase (PEPCK), a key gluconeogenic enzyme, is associated with adaptation to environmental stress. However, its potential role in diapause is not known. Here, two cytoplasmic genes encoding PEPCK (*SmPEPCK1-1* and *SmPEPCK1-2*) in *Sitodiplosis mosellana*, a significant wheat pest undergoing obligatory larval diapause during the third instar, were cloned, and their expression patterns during diapause and thermal stress were assessed, together with the effects of *SmPEPCK1-2* knockdown on larval development. Both *SmPEPCK1-1* and *SmPEPCK1-2* were evolutionarily conserved and contained canonical functional domains and motifs. Their expression was induced by diapause, and was found to be tissue-specific, with the highest levels observed in the fat bodies of diapausing larvae. Furthermore, exposure to heat stress in oversummering larvae or cold stress in overwintering larvae enhanced the expression of both genes within specific temperature ranges (35–40 °C and −10–0 °C, respectively). RNA interference-mediated knockdown of *SmPEPCK1-2* did not affect cocoon-breaking rates and timing but significantly prolonged the duration of larval development from cocoon-breaking to pupation. These findings indicate that both *SmPEPCK* genes are closely involved in tolerance to diapause-related stress, with *SmPEPCK1-2* also contributing to the regulation of larval development.

## Simple Summary

Phosphoenolpyruvate carboxykinase (PEPCK) is a crucial metabolic enzyme involved in gluconeogenesis. However, little is known of its role in the diapause response. In this study, two cytoplasmic genes encoding PEPCK (*SmPEPCK1-1* and *SmPEPCK1-2*) in *Sitodiplosis mosellana* Géhin (Diptera: Cecidomyiidae), a significant wheat pest that undergoes an obligatory diapause during its third-instar larval stage, were cloned, and their expression during diapause and temperature stress was analyzed, together with the effects of *SmPEPCK1-2* knockdown on larval development. The results suggested that the expression of both *SmPEPCK1-1* and *SmPEPCK1-2* is regulated by diapause and the environment, with *SmPEPCK1-2* also involved in the regulation of larval development.

## 1. Introduction

To survive seasonal changes and adverse environmental conditions, many insects have evolved a suite of physiological and behavioral adaptations, of which diapause is one of the most significant [1]. Diapause is an exogenously triggered endogenously regulated developmental arrest, characterized by lowered metabolic activity and increased tolerance of environmental stressors [2,3]. It exists in two types: facultative diapause, which is induced by environmental signals such as photoperiod and temperature; and obligatory diapause, which occurs at a predetermined developmental stage without reliance on external stimuli [4,5,6]. This represents an ecologically important adaptive strategy as it enables alignment of insect life histories with seasonally favorable conditions, thereby influencing species distribution and evolutionary trajectories [7,8]. Therefore, an in-depth understanding of the mechanisms underlying diapause is key to the formulation of effective integrated pest-control strategies in agriculture [9].

Phosphoenolpyruvate carboxykinase (PEPCK) functions as a key enzyme involved in gluconeogenesis by catalyzing the decarboxylation of oxaloacetate to phosphoenolpyruvate, a critical reaction that enables glucose generation from non-carbohydrate sources when food is scarce [10]. PEPCK has two distinct isoforms, namely, the cytosolic (PEPCK-C) and mitochondrial (PEPCK-M) forms, each of which is encoded by a separate nuclear gene (*PCK1* or *PCK2*) [11,12]. The expression of these isoforms is tissue-specific [13,14], although both participate in glucose production [15].

PEPCK enzymes fall into two categories according to their substrate dependence, namely, ATP-dependent and GTP-dependent forms [16]. The GTP-dependent form is found predominantly in animals, while the ATP-dependent form is mainly present in plants and bacteria, with both containing highly conserved domains and motifs [17,18,19]. To date, PEPCK-encoding genes have been isolated and characterized from a wide range of taxa. They play roles in fundamental biological processes, including insect gametogenesis and embryogenesis [14], antiviral immune responses [20], maintenance of cellular redox homeostasis [21,22], and adaptation to environmental stressors (particularly thermal stress and desiccation) [23,24,25]. Despite demonstration of these functions, little is known of the association between PEPCKs and insect diapause [26,27].

The wheat midge *Sitodiplosis mosellana* Géhin (Diptera: Cecidomyiidae) is a devastating insect pest of wheat, causing substantial economic damage during outbreak years [28,29]. The insect produces only one generation each year. In the wheat-growing areas of northern China, adult midges emerge in late April when they oviposit on wheat ears [30]. After hatching, the young larvae feed on the developing cereal grains before molting into the third instar. The mature third-instar larvae move from the wheat spikes in mid-to-late May, migrating downward into the soil where they form larval cocoons associated with obligatory diapause. The larvae then remain within the cocoons for up to nine months until mid-March of the following year. As soil temperatures increase in spring, the larvae exit their cocoons and undergo post-diapause development. This prolonged diapause protects the midges from harsh environmental conditions associated with the changing seasons, enabling alignment of their life cycles with the stages of wheat growth.

To explore the potential function of PEPCKs in *S. mosellana* diapause, two cytoplasmic genes encoding PEPCK (*SmPEPCK1-1* and *SmPEPCK1-2*) were isolated and their expression was analyzed across different diapause stages, tissues, and thermal stress conditions. In addition, RNA interference (RNAi) was utilized to knock down the expression of *SmPEPCK1-2*, and the length of time between cocoon-breaking and pupation was compared between the knockdown and control larvae. These findings assist in clarifying the molecular mechanisms underlying diapause in *S. mosellana*, and provide a reference for the design of innovative control methods against this major pest.

## 2. Materials and Methods

### 2.1. Insect Sourcing and Collection

The third larval instar of *S. mosellana* used for this study were collected from natural habitats as we have described previously [31]. Specifically, pre-diapause larvae were collected by dissection of wheat spikes that had been severely damaged by *S. mosellana* during late May 2024. At the same time, mature wheat ears containing third-stage larvae were harvested and transferred to soil within a specially prepared field insectary at Northwest A&F University (Yangling, China) (34°16′ N, 108°4′ E). Soil moisture was maintained by periodic watering to initiate and terminate diapause, with successful transition into diapause confirmed by the formation of larval cocoons. The cocooned larvae were sampled monthly between 25 June 2024, and 25 February 2025. We have previously found that cocooned larvae collected from December onward successfully undergo eclosion as adults when maintained at 25 °C, indicating that diapause was already terminated and that they had entered post-diapause quiescence by that time [32]. Larvae undergoing post-diapause development (i.e., larvae breaking out of their cocoons) were sampled in mid to late March 2025. All collected samples were immediately snap-frozen in liquid nitrogen before transfer to −80 °C storage for subsequent examination.

### 2.2. RNA Isolation, cDNA Synthesis, and gDNA Extraction

Total RNA was extracted from pre-diapause larvae (n = 20) using TRIzol reagent (TaKaRa, Dalian, China). The quality of the extracted RNA was estimated using 1% agarose gels, while concentrations were measured using a BioSpec-nano spectrophotometer (Shimadzu, Kyoto, Japan). One microgram of the RNA was used for cDNA synthesis using a PrimeScript™ RT Reagent Kit with gDNA Eraser (TaKaRa, Dalian, China). Genomic DNA was extracted from an equivalent pooled larval sample using the Biospin Insect Genomic DNA Extraction Kit (Bioer Technology Co., Ltd., Hangzhou, China), following the provided guidelines.

### 2.3. Cloning of Open Reading Frames

Using a combination of our laboratory-established transcriptomic dataset (data unpublished) and a genomic database of *S. mosellana* [33], specific primers targeting *S. mosellana PEPCK1-1* and *PEPCK1-2* (Table 1) were designed to amplify the respective open reading frames (ORFs) of the genes. Each PCR mixtures (25 μL) comprised 12.5 µL PrimeSTAR Max Premix (Takara, Beijing, China), 1.5 µL cDNA template, 1.0 µL each of upstream and downstream primers, and 9.0 µL ddH_2_O. The PCR protocol consisted of 38 cycles, with denaturation at 98 °C (10 s), annealing at 53 °C (25 s), and extension at 72 °C (40 s). The products were purified on gels and extracted using a gel extraction kit (CWBIO, Beijing, China) before ligation into pEASY^®^-Blunt Zero vectors. The recombinant vectors were transformed into *Trans1-T1* competent cells (TransGen, Beijing, China), yielding positive clones. Three random colonies per amplicon were selected for sequencing by AuGCT DNA-SYN Biotech (Beijing, China) to confirm the accuracy of the inserts.

### 2.4. Bioinformatic Analysis

The molecular weights and isoelectric points of the proteins encoded by *SmPEPCK1-1* and *SmPEPCK1-2* were predicted using the Compute pI/Mw bioinformatics tool (https://web.expasy.org/compute_pi/, accessed on 13 July 2024). Functional domains of PEPCK encoding proteins in *S. mosellana* were searched using the NCBI Conserved Domain Database (https://www.ncbi.nlm.nih.gov/Structure/cdd/wrpsb.cgi, accessed on 13 July 2024). Subcellular localization of the target proteins was predicted using the TargetP 1.1 server (http://www.cbs.dtu.dk/services/TargetP, accessed on 13 July 2024), while DNAMAN v.7.0.2 (Lynnon Biosoft, San Ramon, CA, USA) was utilized for sequence alignment of SmPEPCK1-1 and SmPEPCK1-2 with PEPCK orthologs from other insect taxa. Phylogenetic relationships were examined using the neighbor-joining algorithm implemented in MEGA X version 10.0.1 (Temple University, Philadelphia, PA, USA) with 1000 bootstrap resampling iterations, omitting bootstrap support values below 50.

### 2.5. Temperature Treatments

*Sitodiplosis mosellana* typically survives seasonal environmental extremes by entering diapause as cocooned larvae in the 3–10 cm soil layer [34]. In Yangling District (34°16′ N, 108°4′ E), Shaanxi, China, the maximum temperature in this soil stratum is approximately 35 °C, while the minimum winter temperatures are near 0 °C. However, surface temperatures can frequently surpass 50 °C during summer and drop to −15 °C during winter [35]. To elucidate the potential contributions of *SmPEPCK1-1* and *SmPEPCK1-2* under these conditions, larvae that oversummered and overwintered were exposed to acute thermal stress. At least three independent replicates of each experimental condition were used to ensure reproducibility. Briefly, 20 larvae collected in August or December were placed individually in sterile cryovials. To induce stress responses to heat shock, larvae collected during August were subjected to acute heat stress by immersion in water baths at temperatures between 35 °C and 50 °C for 1 h, with additional larvae (n = 20) exposed to a temperature of 35 °C for varying durations (15–120 min). Similarly, larvae collected in December were placed in temperature-regulated chambers set between −15 °C and 0 °C for 1 h to induce cold stress, with subsets (n = 20) exposed to −10 °C for varying lengths of time (15–120 min). Larvae collected before exposure to temperature changes served as controls. Post-treatment, the larvae were immediately frozen in liquid nitrogen before storage at −80 °C for subsequent examination.

### 2.6. Quantitative Reverse Transcription PCR (qRT-PCR) Analysis

To analyze the expression patterns of *SmPEPCK1-1* and *SmPEPCK1-2* in different tissues, samples of midgut, fat body, and epidermal tissues from a subset of 50 diapausing larvae per group were dissected under a microscope. RNA extraction and subsequent cDNA synthesis were performed as outlined in Section 2.2.

qRT-PCR was used to measure the levels of the two *S. mosellana PEPCK* transcripts and evaluate their responses to diapause and temperature stress. Total RNA was isolated from the different sample cohorts, including larvae at different phases of diapause and temperature-stressed diapausing larvae, followed by reverse transcription to cDNA, as described in Section 2.2. Each 20 µL reaction volume included 10 µL of 2×SuperReal PreMix Plus (Tiangen, Beijing, China), 1.2 µL cDNA template, 0.8 µL each of upstream and downstream primers, and 7.2 µL ddH_2_O. Reactions were run in triplicate on the QuantStudio^®^5 real-time PCR system (Thermo Fisher Scientific, Waltham, MA, USA) with the following program: initial denaturation (95 °C, 30 s), 35 cycles of amplification (95 °C, 5 s; 60 °C, 32 s), and melting curve analysis (95 °C, 15 s; 60 °C, 60 s; 40 °C, 30 s). No-template negative controls in which cDNA was replaced with nuclease-free water were used to verify assay specificity. The internal reference gene for data normalization was *GAPDH* (accession No. KR733066). Each biological sample was analyzed using three technical replicates in three independent experimental runs, The amplification efficiencies of the reference gene (*GAPDH*) and the target genes (*PEPCK1-1* and *PEPCK1-2*) were 103.183%, 103.204%, and 103.195%, respectively. In qPCR analysis, both the double delta Ct and delta Ct calculations are applicable when the amplification efficiencies of the target and reference genes are similar [36,37]. Given the high consistency in gene efficiencies observed in this study, we utilized the widely adopted 2^−ΔΔCt^ method for relative quantification [38,39,40,41].

### 2.7. dsRNA Synthesis and RNA Interference

To generate dsRNA targeting *SmPEPCK1-2* (240 bp) and *GFP* (315 bp as a non-specific negative control), specialized primers containing T7 RNA polymerase promoter sequences at the 5′-termini of both the sense and antisense strands were designed using SnapDragon software (https://www.flyrnai.org/snapdragon, accessed on 13 July 2024). The templates for dsRNA synthesis were obtained using PCR amplification via the T7 RiboMAX Express system (Promega, Madison, WI, USA). Following in vitro transcription, dsRNA integrity was assessed using gel electrophoresis, and concentrations were determined using the BioSpec-nano spectrophotometer (Shimadzu, Kyoto, Japan). The final product was adjusted to a concentration of 10 μg/μL with RNase-free water and stored at −80 °C for further analysis. All procedures were conducted according to the manufacturers’ protocols.

For RNAi assays, larvae within cocoons collected in January, corresponding to the post-diapause quiescent stage, were selected for analysis, due to the high levels of *SmPEPCK1-2* expression observed at this developmental phase (see Section 3). Preliminary in vitro investigations showed that microinjection of 30 nL of 10 µg/µL dsRNA was effective in suppressing the target genes without affecting the viability of the organism. Consequently, the larvae were injected with 300 ng of dsRNA into the intersegmental membrane between segments five and six of the abdomen using a Nanoject II Auto-Nanoliter Injector (Drummond Scientific Company, Broomall, PA, USA). Control cohorts received equivalent volumes of ds*GFP* or DEPC-treated water. After injection, the larvae were incubated on moist filter paper in Petri dishes under standard laboratory settings (24 °C, 70% relative humidity [RH], 16/8 h light/dark [L/D] cycle) for durations of 12, 24, or 48 h. For qRT-PCR analysis, 20 individuals were pooled in each treatment group, totaling three replicates per time point to assess the efficiency of gene silencing.

### 2.8. Effects of SmPEPCK1-2 Knockdown on 3rd Larval Development

After RNAi treatment, the larvae were maintained in an incubator at 24 ± 1 °C with 70 ± 5% RH and a 16:8 h L/D cycle. The larvae were monitored, and the rates and times of cocoon-breaking were recorded daily at fixed time points, and the interval between cocoon breaking and pupation was determined. Each experimental cohort consisted of three biological replicates with 40 individuals per replicate.

### 2.9. Data Analysis

Data on gene expression and developmental parameters were analyzed using one-way ANOVA, with subsequent pairwise comparisons conducted with Tukey’s Honestly Significant Difference (HSD) tests (*p* < 0.05). All data were analyzed using SPSS version 20.0 (SPSS Inc., Chicago, IL, USA), and the results are reported as means ± standard error (SE).

## 3. Results

### 3.1. Characterization of SmPEPCK1-1 and SmPEPCK1-2 cDNA

The amplified ORFs of two *S. mosellana PEPCK* genes, *SmPEPCK1-1* (accession No. PX533515) and *SmPEPCK1-2* (accession No. PX533516), measured 1950 and 2115 bp in length (Figure S1), respectively. The corresponding proteins consisted of 649 and 704 amino acids, with calculated molecular weights of 72.19 and 78.83 kDa and isoelectric points of 6.87 and 8.39. Analysis of both, the cloned cDNA and genomic DNA sequences revealed an absence of introns in both genes.

**Figure 1:**
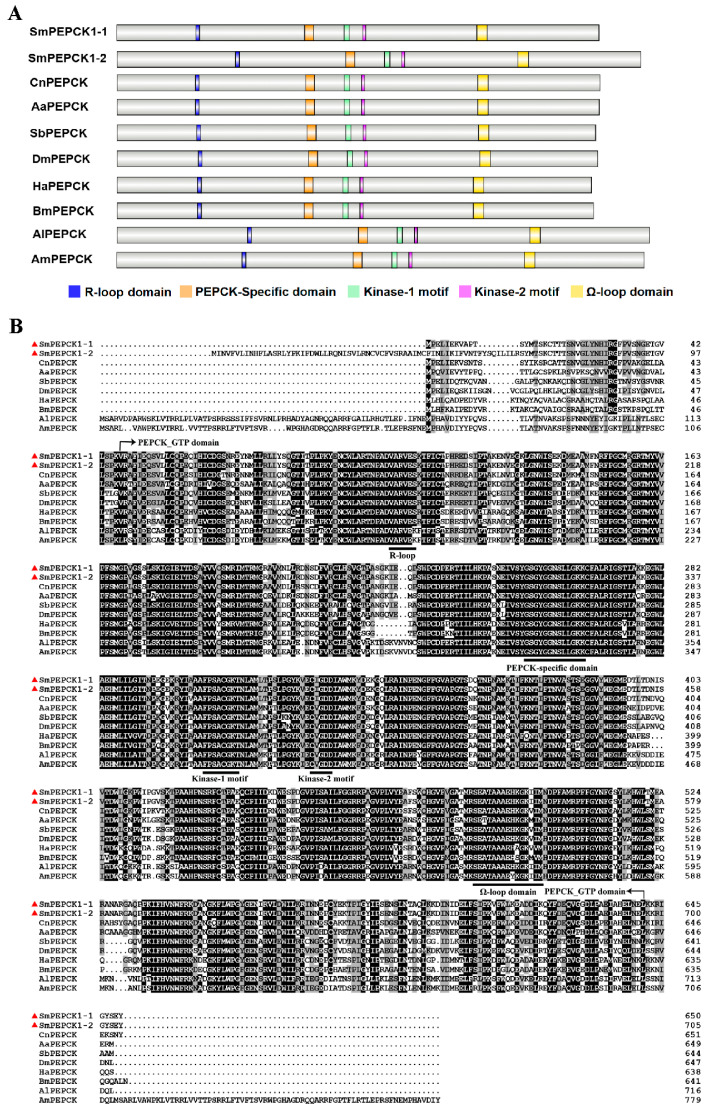
Sequence characterization analysis of PEPCK. (**A**) Schematic diagram of amino acid domains of *Sitodiplosis mosellana* PEPCK (SmPEPCK1-1 and SmPEPCK1-2) (red triangles) and PEPCKs from other insects. (**B**) Multiple alignments of the amino acid sequences of the insect PEPCKs. Black and gray shading corresponds to conserved and similar residues, respectively. The initiator and terminator amino acids of the PEPCK_GTP domain are delineated with arrows. The putative functional domains and motifs are underscored with underlines. The insect taxa and their corresponding NCBI GenBank accession numbers of PEPCKs are listed as follows: *Sitodiplosis mosellana* (SmPEPCK1-1, YBV49471; SmPEPCK1-2, YBV49472), *Contarinia nasturtii* (CnPEPCK, XP_031620284.1), *Aedes aegypti* (AaPEPCK, XP_001647937.2), *Sarcophaga bullata* (SbPEPCK, AYU75340.1), *Drosophila melanogaster* (DmPEPCK, NP_523784.2), *Helicoverpa armigera* (HaPEPCK, AFK28502.1), *Bombyx mori* (BmPEPCK, NP_001040542.1), *Apis laboriosa* (AlPEPCK, XP_043788440.1), and *Apis mellifera* (AmPEPCK, XP_396295.4).

Domain analysis indicated the presence of conserved PEPCK_GTP domains in both SmPEPCKs (a.a. 47–641 in SmPEPCK1-1 and a.a. 102–696 in SmPEPCK1-2) (Figure 1). In addition, key structural characteristics, including the R-loop domain, PEPCK-specific substrate-binding domain, kinase-1 (nucleotide-binding site) and kinase-2 motifs (metal-binding site), and the Ω-loop domain responsible for catalytic activity, were found to be conserved (Figure 1A,B). Predictions of subcellular localization indicated that both proteins were cytoplasmic.

Analysis of homology with other insect proteins indicated the strongest sequence identities between SmPEPCK1-1 (YBV49471) and SmPEPCK1-2 (YBV49472) (85%) with *Contarinia nasturtii* PEPCK (XP_031620284.1), and 66–73% identity with PEPCK orthologs from other insect taxa, including *Aedes aegypti* (XP_001647937.2), *Sarcophaga bullata* (AYU75340.1), *Drosophila melanogaster* (NP_523784.2), *Helicoverpa armigera* (AFK28502.1), *Bombyx mori* (NP_001040542.1), *Apis laboriosa* (XP_043788440.1), and *Apis mellifera* (XP_396295.4). The two SmPEPCKs were 86% identical (Figure 1B).

**Figure 2:**
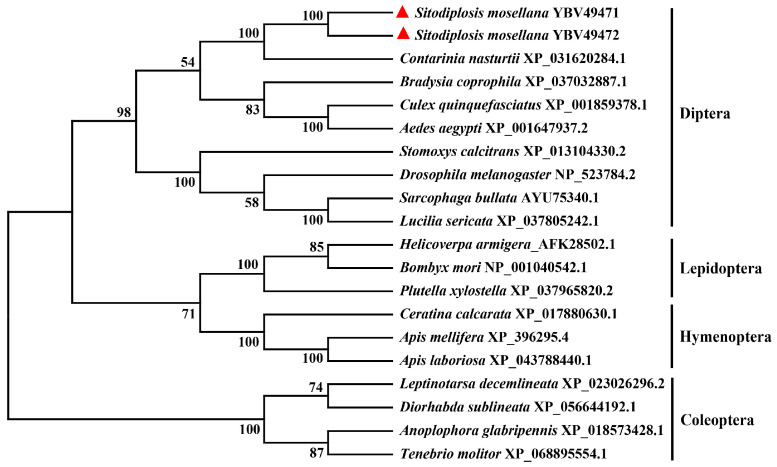
Phylogenetic relationships of SmPEPCK1-1 and SmPEPCK1-2 (red triangles) in relation to other insect PEPCKs based on neighbor-joining algorithm.

Phylogenetic analysis revealed that PEPCKs from Diptera, Lepidoptera, Coleoptera, and Hymenoptera segregated into discrete clades, aligning with their respective taxonomic classifications. Notably, SmPEPCK1-1 and SmPEPCK1-2 exhibited closer phylogenetic affiliations with homologous sequences from the Cecidomyiidae family (particularly *C. nasturtii*), underscoring their close evolutionary relationships (Figure 2).

### 3.2. Expression of SmPEPCK1-1 and SmPEPCK1-2 During Diapause

**Figure 3:**
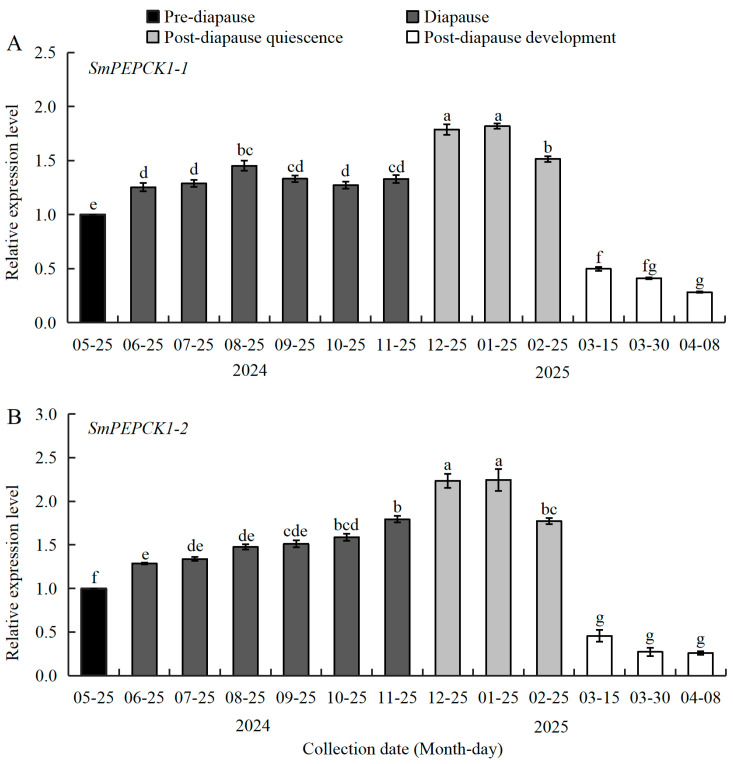
Expression profiles of *SmPEPCK1-1* (**A**) and *SmPEPCK1-2* (**B**) in pre-diapausal, diapausal and post-diapausal stages of *Sitodiplosis mosellana* larvae. Transcript abundance (mean ± SE) of each stage is quantified against pre-diapausal larvae (assigned as 1). Statistically significant differences across diapause stages are indicated by different letters, as determined by Tukey’s multiple range test (*p* < 0.05).

To determine whether the expression of *SmPEPCK1-1*/*PEPCK1-2* correlated with diapause, the mRNA levels of both genes were measured in pre-diapause, diapause, and post-diapause quiescent and post-diapause developmental stages of *S. mosellana* larvae (Figure 3). The abundance of *SmPEPCK1-1* transcripts (*F* = 264.11, df = 12, 26, *p* < 0.05) was significantly increased after diapause entry (June), persisted at raised levels throughout diapause and post-diapause quiescence, and declined significantly thereafter, reaching its lowest level at the post-diapause developmental stage (Figure 3A).

Similarly, the transcriptional levels of *SmPEPCK1-2* (*F* = 156.80, df = 12, 26, *p* < 0.05) increased significantly at diapause onset, after which they continued to rise, peaking during the coldest months of December and January, followed by a marked reduction associated with the resumption of direct development (Figure 3B).

### 3.3. Expression of SmPEPCK1-1 and SmPEPCK1-2 in Different Tissues of Diapausing Larvae

**Figure 4:**
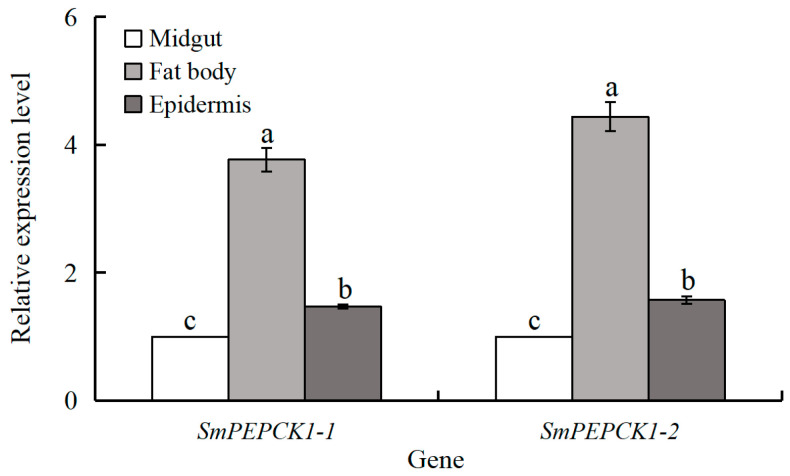
Expression profiles of *SmPEPCK1-1* and *SmPEPCK1-2* at different tissues of diapausing *Sitodiplosis mosellana* larvae. Transcript abundance (mean ± SE) of each tissue is quantified against the midgut (assigned as 1). Statistically significant differences among tissues are indicated by different letters, as determined by Tukey’s multiple range test (*p* < 0.05).

Tissue-specific expression patterns were observed for both *SmPEPCKs* (Figure 4). Both were expressed predominantly in the fat body, with significantly lower transcript levels observed in the midgut and epidermis of third-instar diapausing larvae. Compared to those in the midgut, the expression levels of *SmPEPCK1-1* (*F* = 190.36, df = 2, 6, *p* < 0.05) and *SmPEPCK1-2* (*F* = 248.23, df = 2, 6, *p* < 0.05) were 3.8-fold and 4.4-fold higher in the fat body, and 1.5-fold and 1.6-fold higher in the epidermis, respectively (Figure 4).

### 3.4. Expression of SmPEPCK1-1 and SmPEPCK1-2 in Response to Heat Shock During Diapause

**Figure 5:**
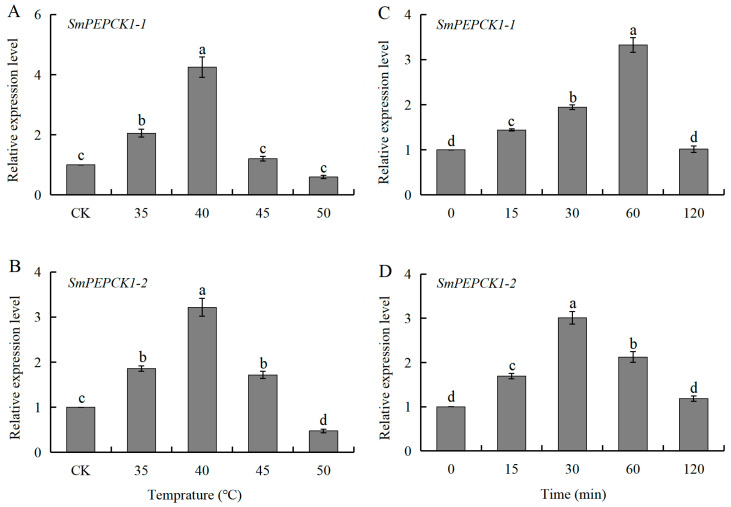
Heat stress-responsive expression patterns of *SmPEPCK1-1* and *SmPEPCK1-2* in oversummering diapausing larvae exposed to different high-temperatures (35–50 °C) for 1 h (**A**,**B**) or to 40 °C for varying lengths of time (0–120 min) (**C**,**D**). Transcript abundance (mean ± SE) of each treatment is quantified against the untreated group (CK, 0 min) (assigned as 1). Statistically significant differences among treatments are indicated by different letters, as determined by Tukey’s multiple range test (*p* < 0.05).

Exposure of oversummering diapausing larvae to raised temperatures above those of their typical habitat for 1 h led to a significant increase in the expression of *SmPEPCK1-1* (*F* = 75.96, df = 4, 10, *p* < 0.05) between 35 and 40 °C, reaching a maximum 4.3-fold increase at 40 °C compared to the controls; however, there was no further upregulation at higher temperatures between 45 °C and 50 °C (Figure 5A). Similarly, *SmPEPCK1-2* (*F* = 105.42, df = 4, 10, *p* < 0.05) expression rose significantly between 35 °C and 45 °C, with a peak 3.2-fold increase at 40 °C, followed by a significant decline at temperatures above 45 °C (Figure 5B).

The transcriptional levels of *SmPEPCK1-1* (*F* = 133.89, df = 4, 10, *p* < 0.05) and *SmPEPCK1-2* (*F* = 76.24, df = 4, 10, *p* < 0.05) were also affected by the duration of treatment. At 40 °C, both transcripts showed significant upregulation after 15 min, reaching peak expression levels at 60 min for *SmPEPCK1-1* and 30 min for *SmPEPCK1-2* (Figure 5C,D).

### 3.5. Expression of SmPEPCK1-1 and SmPEPCK1-2 in Response to Cold Shock During Diapause

**Figure 6:**
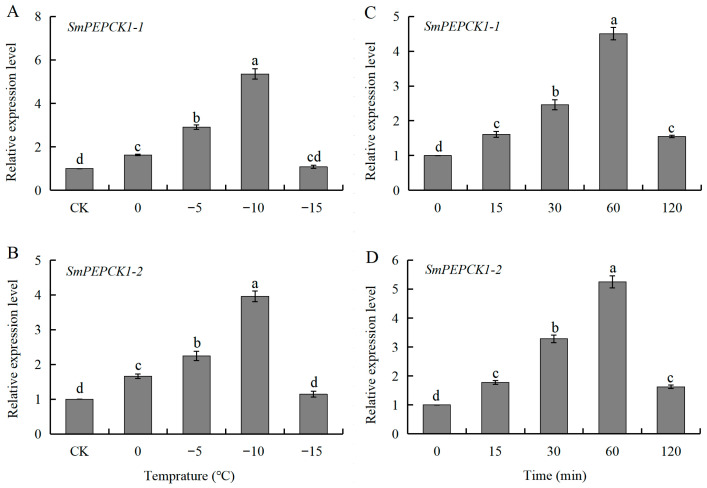
Cold stress-responsive expression patterns of *SmPEPCK1-1* and *SmPEPCK1-2* in overwintering diapausing larvae exposed to different low-temperatures (0–15 °C) for 1 h (**A**,**B**) or to −10 °C for varying lengths of time (0–120 min) (**C**,**D**). Transcript abundance (mean ± SE) of each treatment is quantified against the untreated group (CK, 0 min) (assigned as 1). Statistically significant differences among treatments are indicated by different letters, as determined by Tukey’s multiple range test (*p* < 0.05).

Cold exposure also had marked effects on the expression of *SmPEPCK1-1* (*F* = 236.90, df = 4, 10, *p* < 0.05) and *SmPEPCK1-2* (*F* = 137.52, df = 4, 10, *p* < 0.05) in overwintering diapausing larvae. At temperatures between 0 °C and −10 °C, the expression of both *SmPEPCK1-1* and *SmPEPCK1-2* increased as the temperature decreased, peaking at −10 °C with approximately 5.4-fold and 4.0-fold increases, respectively. At −15 °C, the transcript levels of both genes reverted to baseline levels relative to the controls (Figure 6A,B).

At −10 °C, the expression levels of *SmPEPCK1-1* (*F* = 151.98, df = 4, 10, *p* < 0.05) and *SmPEPCK1-2* (*F* = 215.78, df = 4, 10, *p* < 0.05) were similar to those of the overwintering diapause larvae. The expression of both genes increased markedly over time, with significant upregulation observed at 30 min, reaching a peak at 60 min (Figure 6C,D).

### 3.6. Effects of SmPEPCK1-2 Knockdown on the Duration of Development in Third Instar Larvae

**Figure 7:**
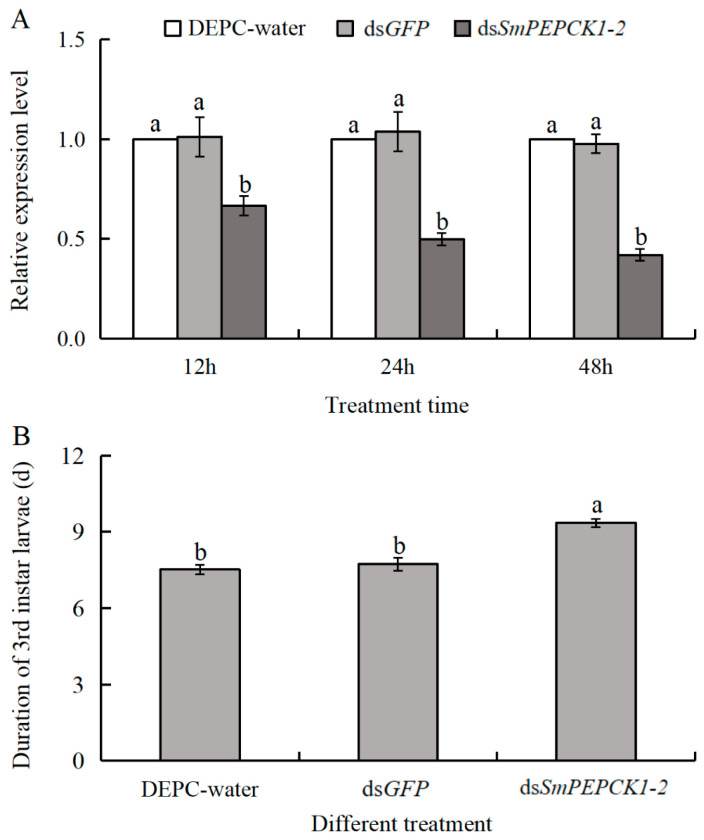
The effect of dsRNA injection on *SmPEPCK1-2* expression (**A**) and its impact on the length of development between cocoon-breaking and pupation in third-instar larvae (**B**). Transcript abundance (mean ± SE) of each time point is quantified against the DEPC-water group (assigned as 1). Statistically significant differences among treatments are indicated by different letters, as determined by Tukey’s multiple range test (*p* < 0.05).

RNAi-mediated knockdown of *SmPEPCK1-2* significantly reduced the expression levels at three distinct time points (12 h: *F* = 9.53, df = 2, 6, *p* < 0.05; 24 h: *F* = 24.52, df = 2, 6, *p* < 0.05; 24 h: *F* = 102.75, df = 2, 6, *p* < 0.05). After treatment with ds*SmPEPCK1-2*, the mRNA levels of *SmPEPCK1-2* were observed to be significantly decreased by 33%, 50%, and 58% at 12, 24, and 48 h, respectively, when compared to the DEPC-treated water (Macklin, Shanghai, China) control. Similarly, reductions of 34%, 52%, and 57% were observed at the same time points relative to the ds*GFP* control group (Figure 7A). These results confirm the effective knockdown of the target gene.

To assess the association between *SmPEPCK1-2* and *S. mosellana* development, we examined key developmental parameters of third-instar larvae after treatment with ds*SmPEPCK1-2*. The results indicated that while the cocoon-breaking rates and timing of the third instar larvae remained unaffected, the interval between cocoon-breaking and pupation was significantly prolonged after treatment with ds*SmPEPCK1-2* (*F* = 17.07, df = 2, 6, *p* < 0.05) (Figure 7B, Table S1), suggesting the involvement of this gene in the development of *S. mosellana*.

## 4. Discussion

Diapause is a prevalent phenological adaptation in many agricultural pests, serving as a vital survival strategy during adverse environmental conditions [42,43]. Exploring the mechanisms underlying diapause provides valuable insights for developing targeted pest management strategies. As a critical metabolic enzyme in gluconeogenesis, PEPCK is associated with diapause regulation and physiological adaptation to environmental stress [44,45,46]. PEPCK isoforms vary among species, with both cytosolic PEPCK-C and mitochondrial PEPCK-M found in *A. aegypti* [14] and *S. bullata* [26], while only single types are observed in *B. mori* (PEPCK-M) [20] and *Rhipicephalus microplus* (PEPCK-C) [47]. In the present study, we cloned and characterized a PEPCK-C form comprising two transcripts (*SmPEPCK1-1* and *SmPEPCK1-2*), examined their expression in response to diapause and thermal stress, and also assessed the effects of *SmPEPCK1-2* knockdown on the length of development between cocoon-breaking and pupation in third-instar larvae. Unlike most other molecular investigations on insect diapause, the insect samples used in this study were collected from all developmental stages from a natural population, which may more accurately reflect natural conditions.

Like other known PEPCK proteins [19,48], the identified SmPEPCKs contained the conserved PEPCK_GTP domain (Figure 1), which provides the structural basis for their GTP-dependent catalysis of the phosphorylation and decarboxylation of oxaloacetate (OAA) to phosphoenolpyruvate [27,49]. Moreover, the structures necessary for catalysis, including the R-loop and PEPCK-specific substrate-binding domain, the nucleotide-binding kinase-1 motif, metal-binding kinase-2 motif, and the Ω-loop domain [19,48,50,51], were observed to be conserved in the SmPEPCKs (Figure 1), indicating their active involvement in gluconeogenic pathways in *S. mosellana*. As both SmPEPCK1-1 and SmPEPCK1-2 contained the conserved structures necessary for OAA and GTP binding, it is inferred that they are GTP-dependent PEPCKs.

Several previous studies have shown that PEPCK activity is regulated transcriptionally, with its expression directly influencing gluconeogenic flux and playing a critical role in the maintenance of energy homeostasis [52,53]. The present investigation of *S. mosellana* PEPCKs revealed significant upregulation of *SmPEPCK1-1* and *SmPEPCK1-2* during diapause, especially in the low-temperature quiescent phase (Figure 3), and were correlated with the changes in glycogen phosphorylase, trehalose synthetic genes and corresponding enzymatic activities [54,55]. These concomitant alterations may contribute to the maintenance of a hypometabolic state during diapause, reducing energetic expenditure and aiding the insect in surviving unfavorable environmental conditions [10]. The PEPCK expression patterns observed here are consistent with those observed in diapausing individuals of *Sarcophaga crassipalpis* [56], *S. bullata* [26], *Trogoderma granarium* [27], and *Arma chinensi s* [57], but differ from those reported in diapausing *Megachile rotundata* [58], *Exorista civilis* [59], and *Aphidoletes aphidimyza* [60]. These variations may reflect evolutionary divergence among species in the regulation of gluconeogenesis to meet energy requirements of post-diapausal development.

The observed tissue-specific expression patterns may reflect differences in the functions of PEPCK proteins [14,20]. Our findings indicated significantly higher expression of *SmPEPCK1-1* and *SmPEPCK1-2* in fat bodies compared with the epidermis and midgut (Figure 4), which is consistent with the general recognition that gluconeogenesis occurs largely in the fat bodies of most arthropods, organs that are functionally equivalent to the mammalian liver and adipose tissue [10,61,62]. Notably, *SmPEPCK1-1*/*PEPCK1-2* expression in the epidermis was also significantly higher than that in the midgut (Figure 4), potentially reflecting the function of the epidermis as the first-line defense against external stressors (e.g., thermal fluctuations), necessitating rapid adaptive responses, although the roles of the genes in the epidermis remain unclear.

Similarly to the stress-responsive expression patterns of *PEPCKs* observed in *D. melanogaster* [63], *S. bullata* [24], and *Belgica antarctica* [64], the two *PEPCK* transcripts in *S. mosellana* were observed to be significantly upregulated in response to acute thermal (heat and cold) stress during larval diapause within a certain range (Figure 5 and Figure 6). This pattern was correlated with changes in sugar alcohols (e.g., trehalose and/or glycerol) in diapausing *S. mosellana* and *Ostrinia furnacalis* at low temperatures [65,66]. Studies have shown that increased expression of *PEPCK* and glycogen phosphorylase (*Gp*) in *B. antarctica* under both cold and heat stress enhances glycogen catabolism and gluconeogenesis, thereby enabling adaptation to harsh environmental conditions [64]. This regulatory pathway appears to be evolutionarily conserved, as shown by similar findings in the overwintering pupae of *S. bullata*, where *PEPCK* upregulation may trigger synthesis of the precursors of cryoprotectants such as sugars and polyols [26]. Parallel observations in *A. chinensis* during cold-induced diapause suggest that *PEPCK* may be involved in the synthesis of antifreeze compounds essential for survival during diapause at low temperatures [57]. It is hypothesized that *S. mosellana PEPCK1-1*/*PEPCK1-2* may perform analogous roles during adaptations to environmental stress. However, this hypothesis requires further biochemical verification, including measurement of enzyme activities and substrates, to clarify its specific metabolic functions. Notably, the expression of *SmPEPCK1-1* and *SmPEPCK1-2* was significantly downregulated beyond the threshold temperature (Figure 5 and Figure 6) indicates that the cellular damage caused by thermal stress has exceeded the stress response capacity of *PEPCK*. This underscores the limited potential of *PEPCK* induction in insects to mitigate cellular injury under extreme thermal stress.

Beyond their roles in stress responses, *PEPCKs* are also integral to the regulation of the growth, development, and lifespan of the organism [67,68]. For instance, in *Mus musculus*, overexpression of *PEPCK*-C has been found to significantly extend lifespan [69]. Similarly, in transgenic *Caenorhabditis elegans*, *PEPCK*-C overexpression resulted in a 22% increase in longevity, while survival was reduced after knockdown [70]. Furthermore, RNAi-mediated knockdown of *PEPCK* in the bloodstream form of *Trypanosoma brucei* led to cell cycle arrest [71]. Due to the absence of highly specific and efficient primers for *SmPEPCK1-1*, the present study focused exclusively on *SmPEPCK1-2*. The results showed that *SmPEPCK1-2* knockdown had no effect on either cocoon-breaking rates or timing in *S. mosellana* larvae, although it significantly prolonged larval development (Figure 7B), suggesting that *SmPEPCK1-2* plays a critical role in the larval development of this pest species. This may result from *PEPCK* suppression disrupting gluconeogenesis, which could reduce glucose production and cause pyruvate accumulation, potentially slowing the tricarboxylic acid cycle and weakening energy metabolism [72].

## 5. Conclusions

The findings of this study suggest that upregulation of *SmPEPCK1-1* and *SmPEPCK1-2* induced by both diapause and acute thermal stress likely plays a crucial role in mediating diapause-related responses to environmental stress. Moreover, *SmPEPCK1-2* is involved in the regulation of larval development in *S. mosellana*. Further investigation into the physiological functions of *SmPEPCK1-2* during development could lead to improved strategies for controlling this insect pest.

## Figures and Tables

**Table 1:** Primer sequences used in this study.

Primer Name	Sequence (5′ to 3′)	Purpose
PEPCK1-1 sense	AGCGAAATAAAAATGCCAG	ORF and gDNA cloning
PEPCK1-1 antisense	CTACATGATCCAATCCAGC	
PEPCK1-2 sense	GCTTACACTTTGTGATTGCAG	
PEPCK1-2 antisense	CCGATTGATCCCCTTATTCG	
dsPEPCK1-2 sense	taatacgactcactatagggTTCTGTGCTCCGAAACTGTG	dsRNA synthesis
dsPEPCK1-2 antisense	taatacgactcactatagggGATTGCTCAATGAATGCACG	
dsGFP sense	taatacgactcactatagggGTGTTCAATGCTTTTCCCGT	
dsGFP antisense	taatacgactcactatagggCAATGTTGTGGCGAATTTTG	
PEPCK1-1 sense	GCCAGAACTCATCGAAAAAGTTG	qPCR
PEPCK1-1 antisense	CGCACTTTTGGCGATAGAAC	
PEPCK1-2 sense	GATTGGCTGCTCCGTCAGA	
PEPCK1-2 antisense	CGTCTCTCCGTTTGACACC	
GAPDH sense	CCATCAAAGCAAGCAAGA	
GAPDH antisense	CAGCACGGAGCACAAGAC	

## Data Availability

The original contributions presented in this study are included in the article/Supplementary Material. Further inquiries can be directed to the corresponding authors.

## Supplementary Materials

The following supporting information can be downloaded at: https://www.mdpi.com/article/10.3390/biology15020147/s1, Figure S1: Nucleotide and deduced amino acid sequences of *Sitodiplosis mosellana* PEPCKs (SmPEPCK1-1 and SmPEPCK1-2). The initiation and termination codons are indicated with ellipses. PEPCK_GTP domain are delineated with grey shading. The structures necessary for catalysis, including the R-loop domain (V105-S110 for SmPEPCK1-1 and V160-S165 for SmPEPCK1-2), the PEPCK-specific domain (G253-K265 for SmPEPCK1-1 and G308-K320 for SmPEPCK1-2), the Kinase-1 motif (F305-T312 for SmPEPCK1-1 and F360-T367 for SmPEPCK1-2), the Kinase-2 motif (C328-D332 for SmPEPCK1-1 and C383-D387 for SmPEPCK1-2), and the Ω-loop domain (S484-M498 for SmPEPCK1-1 and S539-M553 for SmPEPCK1-2), are underscored with boxes; Table S1: Developmental parameters of cocooned larvae injected with dsRNAs.
