# Supplementary material for: Characterization and Expression of Two Cytoplasmic Phosphoenolpyruvate Carboxykinase Genes Associated with Larval Diapause and Temperature Stress in the Wheat Blossom Midge, Sitodiplosis mosellana
Source: Biology (Basel). 2026 Jan 14;15(2):147. doi: 10.3390/biology15020147 (PMC12837266; doi:10.3390/biology15020147)
Supplement: Supplementary file 1 [file biology-15-00147-s001.zip › biology-4026883-supplementary.pdf]

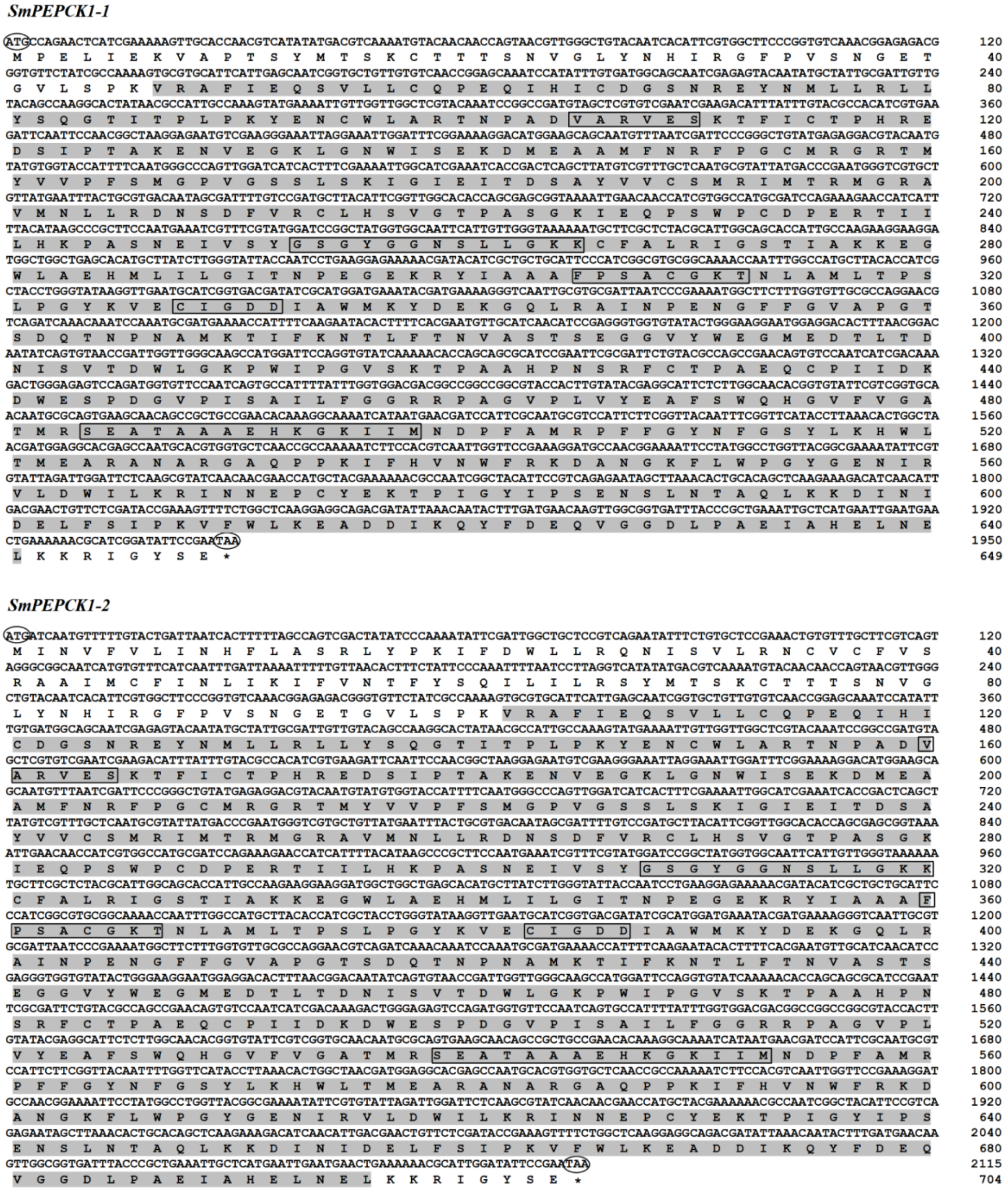

Figure S1. Nucleotide and deduced amino acid sequences of *Sitodiplosis mosellana PEPCKs* (*SmPEPCK1-1* and *SmPEPCK1-2*). The initiation and termination codons are indicated with ellipses. PEPCK_GTP domain are delineated with grey shading. The structures necessary for catalysis, including the R-loop domain (V105-S110 for SmPEPCK1-1 and V160-S165 for SmPEPCK1-2), the PEPCK-specific domain (G253-K265 for SmPEPCK1-1 and G308-K320 for SmPEPCK1-2), the Kinase-1 motif (F305-T312 for SmPEPCK1-1 and F360-T367 for SmPEPCK1-2), the Kinase-2 motif (C328-D332 for SmPEPCK1-1 and C383-D387 for SmPEPCK1-2), and the Ω-loop domain (S484-M498 for SmPEPCK1-1 and S539-M553 for SmPEPCK1-2), are underscored with boxes.

Table S1 Developmental parameters of cocooned larvae injected with dsRNAs

**Larval instar**	**Treatments**	**Cocoon-breaking rate**	**Cocoon-breaking timing (day)**
3rd instar	DEPC-water	0.8833±0.0221	1.8087±0.0085
	ds*GFP*	0.8500±0.038	1.7110±0.0730
	ds*PEPCK1-2*	0.8417±0.0167	1.6800±0.0810

The data were the mean ± standard error (SE) of three biological replicates. No significant difference was observed between the treatment and the control groups (Tukey's multiple range test, $P < 0.05$).
